# Timely Surgical Resection Achieved Prolonged Remission in Patients With Malignant Mesothelioma of the Tunica Vaginalis Testis and Retroperitoneal Recurrence

**DOI:** 10.7759/cureus.59052

**Published:** 2024-04-26

**Authors:** Milan Radovanovic, Uros Kojic, Aleksa Zubelic, Djordje Jakulic, Zoran Filipovic

**Affiliations:** 1 Clinic of Urology, University Clinical Centre of Serbia, Clinic of Urology, Belgrade, SRB; 2 Urology, University Hospital Medical Center Bezanijska Kosa, Belgrade, SRB; 3 Urology, Varisano Klinik Bad Soden, Bad Soden, DEU

**Keywords:** surgery, remission, rplnd, testicular tumor, malignant mesothelioma

## Abstract

Malignant mesothelioma of the tunica vaginalis testis (MMTVT) is a rare tumor of the testicular sheath. More than 50% of cases initially present as recurrent hydrocele, but there have also been documented cases with hematocele, inguinal hernia, or epididymitis. Due to the non-specific symptoms and signs of the disease, it is almost always diagnosed intra- or postoperatively. The lack of standardized therapy protocols, as well as the lack of evidence supporting systemic chemotherapy, have fueled arguments in favor of the necessity of retroperitoneal lymph node dissection (RPLND) in the treatment of the disease, especially in the case of lymph node metastasis. We present a case of MMTVT achieving prolonged remission after timely and extensive surgical treatment.

## Introduction

Malignant mesothelioma of the tunica vaginalis testis (MMTVT) is a rare tumor of the testicular sheath. This malignancy has been reported in less than 400 cases worldwide, and metastatic disease has been described in less than 90 reports [1]. It is most often detected between the ages of 55 and 75, and in older patients, it is associated with a worse prognosis [2]. It is characterized by nonspecific symptoms, with recurrent hydrocele being the most consistent, with a frequency of over 40%. Atypical presentations, including hematocele, inguinal hernia, or epididymitis, have also been described. Although the pathophysiology is unclear, some of the environmental factors with which the occurrence of malignant mesothelioma (MM) is more common can be distinguished, such as long-term exposure to asbestos. In contrast to pulmonary MM, studies have shown that only 30-40% of patients report a history of asbestos exposure [3]. Several other risk factors have been described, including a history of testicular trauma and inflammatory processes in the inguinal region [1]. Considering the appearance of non-specific symptoms and signs, the diagnosis is almost exclusively established intra- or postoperatively. Therapeutic principles are similar to the treatment of pleural or peritoneal mesotheliomas and may include a combination of surgery, radiation therapy, and chemotherapy [1,4]. The lack of standardized therapy protocols results in a variable prognosis that ranges between months and years, as described in different studies, with a median survival described so far of about 23 months [2]. We present an atypical case of MMTVT initially presenting as hydrocele accompanied by hematocele treated with a radical surgical approach, including hemiscrotectomy and timely retroperitoneal lymph node dissection (RPLND) after suspected retroperitoneal recurrence.

## Case presentation

**Figure 1:**
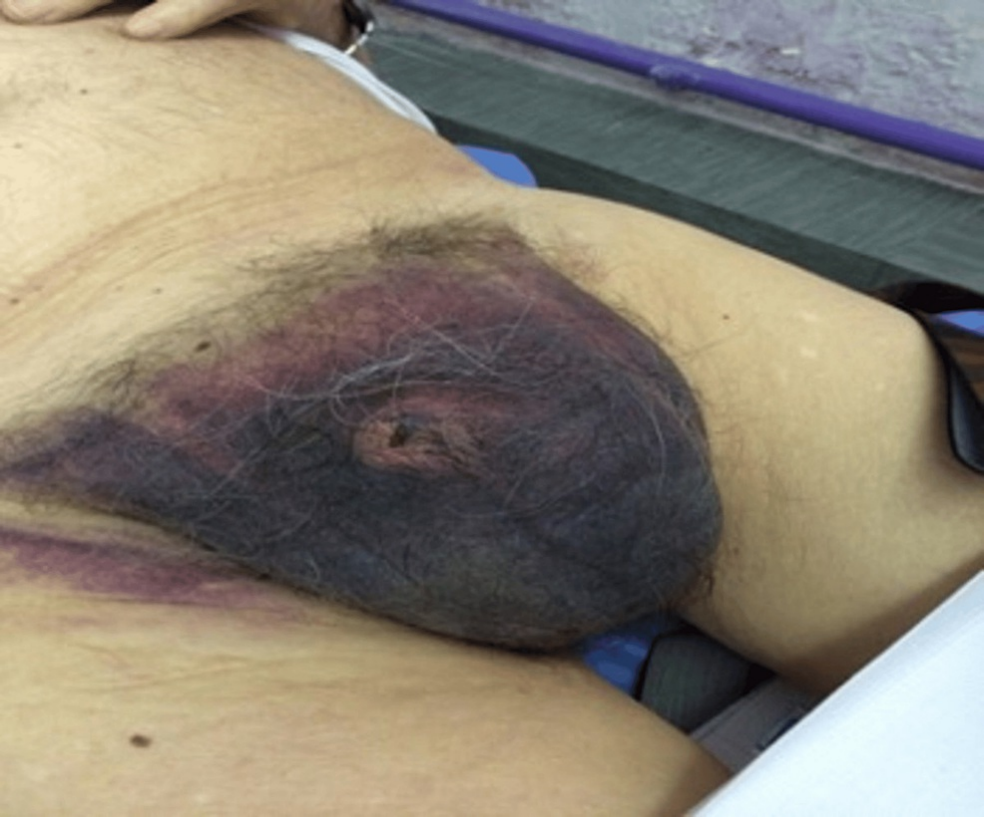
Clinical examination showing scrotal hematoma.

A 59-year-old patient presented himself for examination due to a significant, painful swelling of the scrotum on the right side accompanied by a scrotal hematoma as a result of a minor injury in the genital area approximately seven days before that (Figure 1).

**Figure 2:**
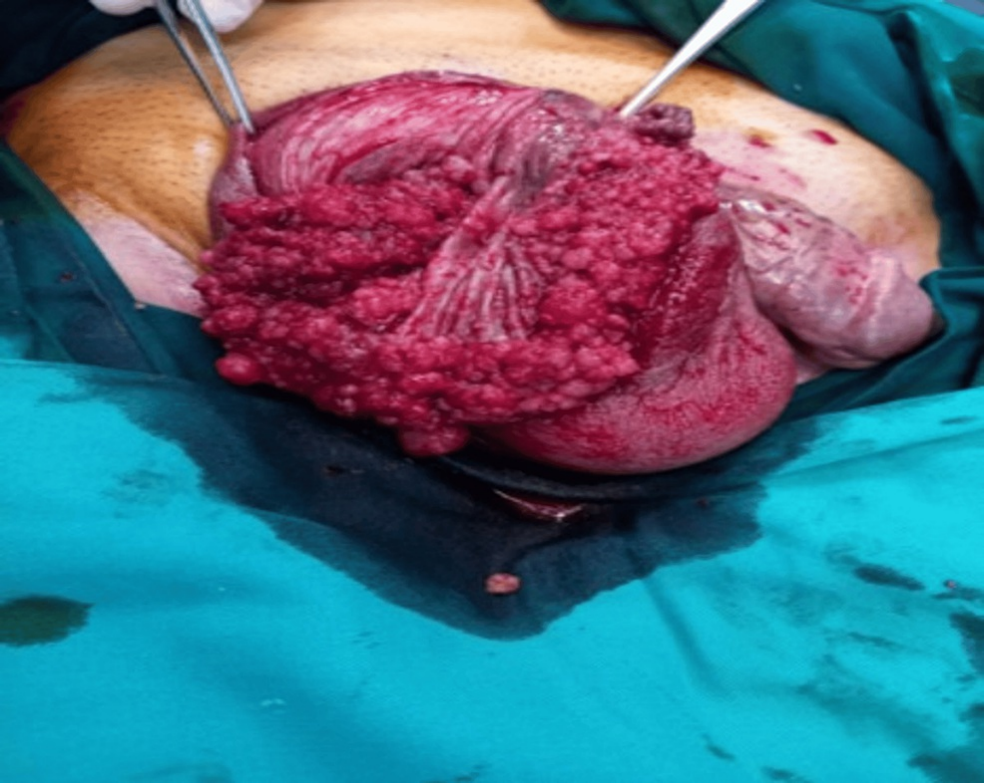
Intraoperative findings: tumor-altered testicular tissue in the form of segments lying on its own neurovascular elements.

During the anamnesis, the patient shared information about the known untreated hydrocele on the right side that first occurred around a year ago and also that he is on permanent anticoagulant therapy - providing early insight into the possible pathophysiology behind scrotal hematoma after a minor injury. A physical examination revealed an enlarged right scrotal sac measuring almost 10 cm, accompanied by a scrotal hematoma. The right testicle was not palpable due to swelling. An ultrasound of both scrotal sacs followed, and it showed that the right scrotal sac was filled with cloudy fluid, probably a hematocele, while the testicular tissue could not be detected as a solid mass but rather as a number of smaller parenchymal heteroechoic parts surrounded by the described fluid. Although the value of tumor markers was within the reference values (AFP, bHCG), due to suspicious sonographic findings, the decision was made to explore the right scrotal sac surgically. Tumor-altered testicular tissue in the form of segments lying on its own neurovascular elements was found intraoperatively, indicating the possible malignant nature of the tissue (Figure 2).

**Figure 3:**
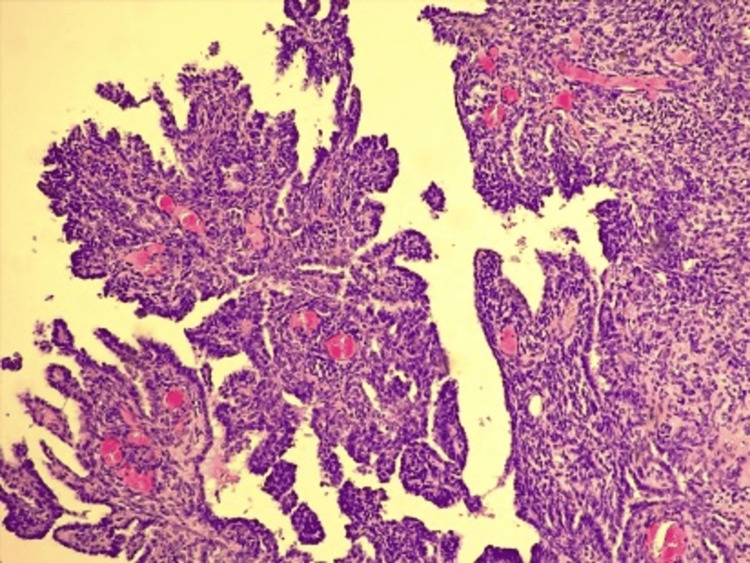
Pathohistological examination showing the malignant mesothelioma of tunica vaginalis testis of epithelioid subtype (CK AE1/AE3 ++ diffuse, WT-1 +++ diffuse, D2-40++ diffuse, CK5/6+ focal, EMA+ focal, Calretinin+ (rare individual cells), T TF1-mCEA). Magnification level 5×.

After that, the decision was made to perform a radical orchiectomy with a hemiscrotectomy on the right side. There were no suspicious lymph nodes, so a lymphadenectomy was not initially performed. Based on morphological and immunohistochemical characteristics, the tumor was classified as malignant mesothelioma of the tunica vaginalis testis of the epithelioid subtype (Figure 3).

**Figure 4:**
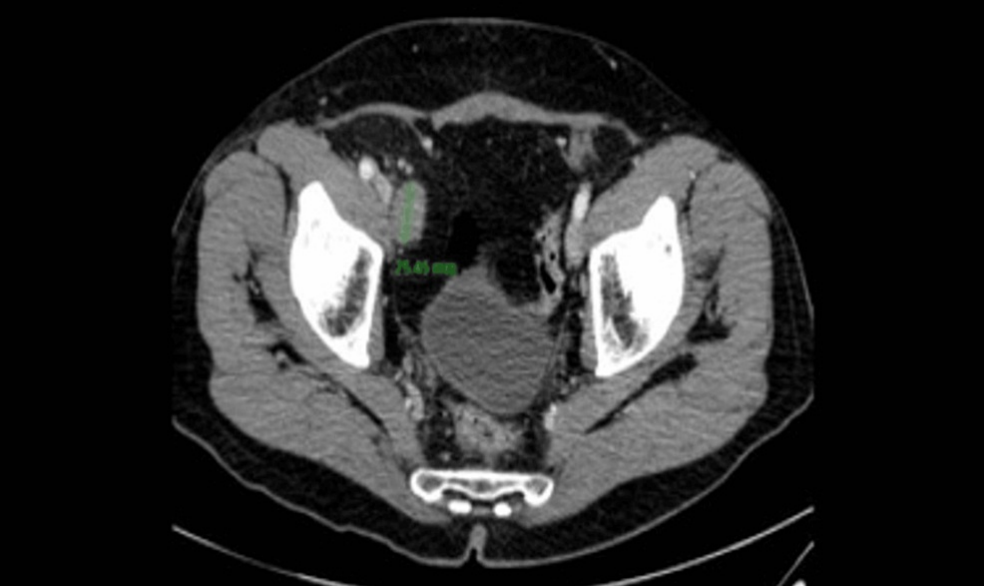
Control NMR of the abdomen and pelvis revealing the presence of enlarged lymph nodes in the iliac region on the right side.

In accordance with the last published data and knowing about the high metastatic potential of MMTVT, computed tomography (CT) of the abdomen and pelvis was performed for the purpose of staging. CT did not reveal enlarged lymph nodes or signs of disease dissemination to other organs. Considering the lack of significant evidence supporting systemic therapy coupled with pathohistological negative resection margins and no evidence of disease dissemination on CT, active imaging surveillance was initiated with follow-up at three months in accordance with similar cases described in the literature [1]. The patient was regularly monitored by imaging methods until 18 months after the initial operation, when control NMR of the abdomen and pelvis revealed the presence of enlarged lymph nodes in the iliac region on the right side (Figure 4).

**Figure 5:**
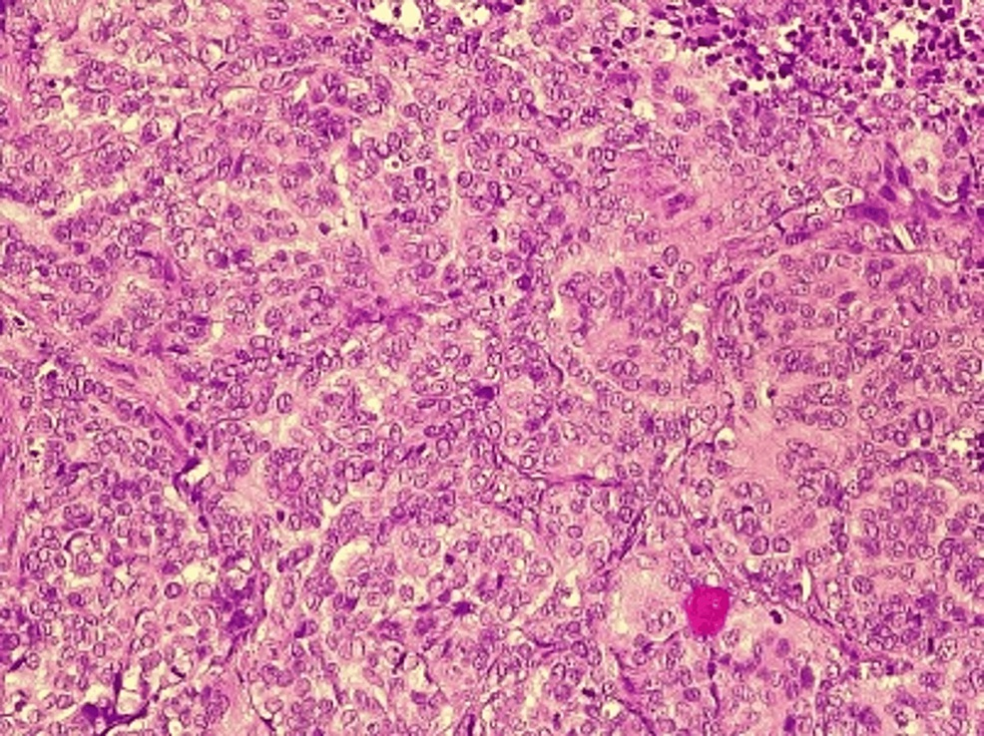
Pathohistological and immunohistochemical analysis confirming lymph node metastasis (CK AE1/AE3 ++ diffuse, WT-1 +++ diffuse, D2-40++ diffuse, CK5/6+ focal, EMA+ focal, Calretinin+ (rare individual cells), T TF1-mCEA). Magnification level 10×.

In light of the results of recently published similar reports indicating that aggressive adjuvant surgery can lead to prolonged remission [5], it was decided to perform timely RPLND in a full bilateral pattern. Pathohistological and immunohistochemical analysis confirmed disease recurrence (Figure 5).

A follow-up of 12 months showed a complete remission of the disease. The patient is currently further under active surveillance.

## Discussion

MMTVT is a very rare tumor and is often mistaken for various benign pathologies of the scrotum. Most cases are described between the ages of 55 and 75, while 10% of the cases are seen in patients younger than the age limit [5,6]. Since Barbera and Rubino first reported about MMTVT in 1957, the total number of described cases in international literature has not exceeded 400 to this day. Asbestos exposure is regarded as a major risk factor for the development of pleural and peritoneal mesotheliomas [4]. However, the link between asbestos and MMTVT is still not sufficiently researched. The study conducted by Bisceglia et al. showed an association with asbestos exposure in only 30-40% of all cases [7]. Several other risk factors have been associated with the development of this malignancy, including various inflammatory processes of the inguinal region and testicular trauma [8]. Early detection of MMTVT remains diagnostically challenging with its inconspicuous onset and very often non-specific manifestation that mimics various other gonadal pathologies, such as a hydrocele [1]. In our case, the most common manifestation, including hydrocele, was accompanied by hematocele and scrotal hematoma as a result of minor trauma under anticoagulant therapy. Studies suggest that more than 95% of patients receive the right diagnosis only after a postoperative pathological examination [9]. The first insight into a possible right diagnosis is the intraoperative discovery of a bloody effusion, papillary mass, or fibrous thickening of the tunica vaginalis testis; similar findings intraoperatively also occurred in our case.

There are three common histological types of malignant mesotheliomas: epithelioid, sarcomatoid, and biphasic [10]. Paratesticular malignant mesothelioma is most often the epithelioid histological subtype (approximately 75% of cases), including ours. A small number of cases of MMTVT result in practically nonexistent treatment guidelines, with radical surgical resection being the first choice for treating paratesticular malignant mesotheliomas [9]. Studies suggest that an aggressive approach with hemiscrotectomy with or without inguinal lymphadenectomy can reduce the risk of recurrence. Thus, that's what we performed on our patient [1,5]. Inguinal lymph node dissection should be performed in patients with suspected lymph node metastasis, which was initially not the case in our patient. The chemotherapy and radiotherapy protocols that are commonly used for the treatment of pleural mesothelioma have also been suggested for MMTVT, especially when presented with lymph node metastasis [11]. The use of radiotherapy remains a topic of discussion. However, studies show that it could be beneficial in preventing disease recurrence following margin-positive surgical resection [8]. A combination of pemetrexed and cisplatin has been a standardized protocol but often proves to be ineffective and requires trying second-line treatment with gemcitabine [4]. Based on negative surgical margins and a lack of sufficient evidence supporting the use of chemotherapy, we decided against it and proceeded with active surveillance. Lymphatic drainage pathways from the testicular region make retroperitoneal lymph nodes the central site for metastatic spread. In our case with NMRT, suspected nodal metastases first occurred 18 months after the initial treatment. RPLND is a standardized modality of treatment in the management of testicular germ cell tumors with suspected retroperitoneal progress; however, this approach is described in less than 15 cases of MMTVT. Despite lacking extensive literature data, we decided to perform RPLND instead of trying questionably effective chemotherapy or radiotherapy in order to reduce morbidity. Following RPLND and pathological examination, we confirmed the spread of lymphatic metastatic disease. Twelve-month follow-up showed total disease remission, showing promising results in this approach and also being in accordance with the last reports on MMTVT [1,12]. In contrast, Grogg et al. showed that patients treated only with RPLND who did not receive additional chemotherapy or radiotherapy progressed again with time, but these results are based on retrospective case reports and small case series. Thus, larger and more consistent datasets are needed to develop prediction models and better define therapy protocols [13].

## Conclusions

Diagnosis of MMTVT remains a challenge given its rarity and variable clinical presentation; nonetheless, it should always be considered in the differential diagnosis of testicular masses. Small case series and the lack of standardized therapy protocols are resulting in a wide array of treatment strategies, often accompanied by a variable prognosis. Our case gives another insight into the possible benefits of timely surgical resection with RLPND in patients with MMTVT and retroperitoneal recurrence, achieving prolonged remission while potentially maintaining lower morbidity by avoiding often ineffective chemotherapy and radiotherapy.
